# Individual‐level drivers of dietary behaviour in adolescents and women through the reproductive life course in urban Ghana: A Photovoice study

**DOI:** 10.1111/mcn.13412

**Published:** 2022-08-08

**Authors:** Julia Liguori, Rebecca Pradeilles, Amos Laar, Francis Zotor, Akua Tandoh, Senam Klomegah, Hibbah A. Osei‐Kwasi, Agnès Le Port, Nicolas Bricas, Richmond Aryeetey, Robert Akparibo, Paula Griffiths, Michelle Holdsworth

**Affiliations:** ^1^ UMR MoISA (Montpellier Interdisciplinary centre on Sustainable Agri‐Food Systems) CIRAD, CIHEAM‐IAMM, INRAE, Institut Agro Montpellier, IRD Montpellier France; ^2^ Centre for Global Health and Human Development, School of Sport, Exercise and Health Sciences Loughborough University Loughborough UK; ^3^ Department of Population, Family & Reproductive Health, School of Public Health University of Ghana Accra Ghana; ^4^ Department of Family and Community Health, School of Public Health University of Health and Allied Sciences Ho Ghana; ^5^ Department of Geography University of Sheffield Sheffield UK; ^6^ School of Health and Related Research University of Sheffield Sheffield UK

**Keywords:** adolescent, behaviours, diet, Ghana, Photovoice, urban, women of childbearing age

## Abstract

Evidence on the individual‐level drivers of dietary behaviours in deprived urban contexts in Africa is limited. Understanding how to best inform the development and delivery of interventions to promote healthy dietary behaviours is needed. As noncommunicable diseases account for over 40% of deaths in Ghana, the country has reached an advanced stage of nutrition transition. The aim of this study was to identify individual‐level factors (biological, demographic, cognitive, practices) influencing dietary behaviours among adolescent girls and women at different stages of the reproductive life course in urban Ghana with the goal of building evidence to improve targeted interventions. Qualitative Photovoice interviews (*n* = 64) were conducted in two urban neighbourhoods in Accra and Ho with adolescent girls (13–14 years) and women of reproductive age (15–49 years). Data analysis was both theory‐ and data‐driven to allow for emerging themes. Thirty‐seven factors, across four domains within the individual‐level, were identified as having an influence on dietary behaviours: biological (*n* = 5), demographic (*n* = 8), cognitions (*n* = 13) and practices (*n* = 11). Several factors emerged as facilitators or barriers to healthy eating, with income/wealth (demographic); nutrition knowledge/preferences/risk perception (cognitions); and cooking skills/eating at home/time constraints (practices) emerging most frequently. Pregnancy/lactating status (biological) influenced dietary behaviours mainly through medical advice, awareness and willingness to eat foods to support foetal/infant growth and development. Many of these factors were intertwined with the wider food environment, especially concerns about the cost of food and food safety, suggesting that interventions need to account for individual‐level as well as wider environmental drivers of dietary behaviours.

## Key messages

- Time constraints, eating at home, eating out, cooking skills, food preferences and food safety concerns were identified as key factors influencing dietary behaviours at the individual level in urban Ghana.
- Ability to eat nutritious, safe food was largely mediated by income and wealth.
- Biological factors, such as pregnancy/lactating status influenced behaviours through medical advice, nutrition knowledge and willingness to promote foetal/infant development.
- Many individual‐level factors were intertwined with the wider food environment.
- Factors influencing dietary behaviour at different levels need to be considered together when developing interventions/policies for healthier diets.

## Graphical abstract

The aim of this study was to identify individual‐level factors influencing dietary behaviours among adolescent girls (13–14 years) and women of reproductive age (15–49 years) in urban Ghana with the goal of building evidence to improve targeted interventions. Qualitative Photovoice interviews (n= 64) were conducted in two urban neighbourhoods in Accra and Ho. Thirty‐seven factors, were identified as having an influence on dietary behaviours: biological (*n* = 5), demographic (*n* = 8), cognitions (*n* = 13) and practices (*n* = 11) with income/wealth (demographic); nutrition knowledge/preferences/risk perception (cognitions); and cooking skills/eating at home/time constraints (practices) emerging most frequently, thus meriting consideration when planning interventions.

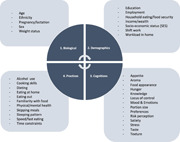

## 1 INTRODUCTION

Sub‐Saharan Africa is rapidly urbanizing and is experiencing changing dietary behaviours as food habits and food environments become increasingly linked to marketization, industrialization and globalized food supplies (Agyemang et al., 2016; Holdsworth & Landais, 2019; Rousham et al., 2020). Changing nutrition landscapes, often referred to as the nutrition transition, have shifted the global disease burden from communicable to non‐communicable diseases (NCDs) (Baker et al., 2020). In 2021, estimates indicated that 77% of NCDs were found in low‐ and middle‐income countries (LMICs) (World Health Organization [WHO], 2021). Increased prevalence of NCDs in these settings is further compounded by multiple burdens of malnutrition (micronutrient deficiencies, undernutrition as well as overweight and obesity), often present within the same individual, household or population (Popkin et al., 2020). Unhealthy diets, propelled by shifts in food environments and dietary changes, are one of the major drivers of this emerging phenomenon. Adolescent girls and women in Africa are more vulnerable to overweight/obesity than men and adolescent boys (Case & Menendez, 2009; Kanter & Caballero, 2012; Muthuri et al., 2014), partly because of the consumption of energy‐dense, nutrient‐poor foods (Sedibe et al., 2014; Trubswasser et al., 2020).

Ghana is a highly urbanized country (~60% of the population lives in urban areas) (Ghana Statistical Services [GSS], 2021) that has reached an advanced stage of the nutrition transition (Agyemang et al., 2016; Ecker & Fang, 2016). Dietary behaviours in urban Ghana have been modified by urbanized lifestyles and increased preference for imported food (Food and Agriculture Organization [FAO], 2009, 2021), which may contribute to the increased prevalence of overweight/obesity among women (34.4% in 2006 to 39.2% in 2016) and school‐aged and adolescent girls (12.6% in 2006 to 17.5% in 2016) (Global Nutrition Report, 2021). NCDs account for 43% of total deaths in Ghana (WHO, 2018, 2020). In addition, poor health outcomes from diet‐related NCDs (DR‐NCDs) are particularly common among Ghanaian women (Agyei‐Mensah & de‐Graft Aikins, 2010; GSS, 2015; Ofori‐Asenso et al., 2016; Ofori‐Asenso et al., 2017). Given this nutritional context, identifying factors that drive dietary behaviours is essential, especially as the Ministry of Health (MoH) of Ghana has placed integrated interventions to promote healthy diets at the core of its public health policies (MoH, 2012, 2020).

A range of models and frameworks have been developed to understand the drivers of food choice and how food environments can influence individual‐level dietary behaviours (Marijn Stok et al., 2018; Osei‐Kwasi et al., 2021; Story et al., 2008; Turner et al., 2018). This paper will contribute to the growing evidence on the influence of individual‐level factors on dietary behaviours in Africa across the different dimensions of the food environment. Individual‐level factors are important to investigate as they may influence food consumption through different pathways, such as self‐efficacy and skills (Story et al., 2008). As adolescent and young adult populations increase worldwide (Norris & Richter, 2016), alongside rapidly changing food environments (Holdsworth & Landais, 2019; Turner et al., 2018), interventions targeting adolescent girls and women of reproductive age are needed as they have the potential to promote positive lifelong and intergenerational nutrition outcomes (Norris et al., 2022; Wells et al., 2020) as they progress into different stages of the reproductive life course. Ensuring good nutrition among all these age groups, coupled with female empowerment, can help improve dietary diversity and overall diet quality in Ghana (FAO, 2021).

This study, therefore, aims to identify the individual‐level drivers of (un)healthy dietary behaviours of adolescent girls and women at different time points during their life course, among socioeconomically deprived urban neighbourhoods in Ghana. More specifically, the study investigates (i) the individual‐level (biological, demographic, cognitions, practices) drivers of (un)healthy food consumption and (ii) whether there are any differences in the factors influencing dietary behaviours between women at different stages of life course (i.e., early adolescence, pregnancy or lactating status).

## 2 METHODS

### 2.1 Study setting

This study was part of a wider project, the *Dietary Transitions in Ghana project* (datalink: https://dataverse.ird.fr/dataverse/diet_trans_ghana;jsessionid=d8c3c605c1c1bf3125e01476d0f6), conducted in Accra (Greater Accra region) and Ho (Volta region), as we were interested in capturing cities with different levels of urbanization and prevalence of overweight/obesity (as a proxy for nutrition transition). In 2015 (study conception), overweight/obesity prevalence among women of reproductive age (WRA) was 57.3% and 31.1% in Greater Accra and the Volta region, respectively (GSS, 2015).

### 2.2 Study design

A qualitative study was conducted among young adolescent girls (13–14 years) and WRA (15–49 years) living in socioeconomically disadvantaged neighbourhoods in Accra and Ho. The study was designed to identify a range of factors at the individual, social, physical and macro‐levels that influence dietary behaviours (Story et al., 2008). This paper reports the findings on the individual‐level factors that emerged. The findings on the influence of the physical‐level (accessibility, affordability, convenience, etc.) food environment on dietary behaviours have been previously published (Pradeilles et al., 2021).

Photovoice, a community‐based participatory photography method, was used to allow participants to document influences on their dietary behaviours in their daily lives. This method facilitates in‐depth exploration, stimulates reflection and enables discussion among participants and policymakers to foster change in a community (Wang, 1999). While Photovoice has largely been used in high‐income countries (Belon et al., 2016; Díez et al., 2017; Gravina et al., 2020; Heidelberger & Smith, 2016), recent studies have used this method in Africa, to assess factors influencing adolescents' dietary behaviours in urban Ethiopia (Trubswasser et al., 2020), among women in rural/urban Uganda (Auma et al., 2020) and balancing work and childcare in Kenya (Hani Sadati et al., 2019). The Photovoice methodology was selected as it places the research participant at the centre of the research process, opening up a pathway for dialogue between the researchers and the participants in a way that face‐to‐face interviews or focus group discussions alone do not. Photographs allow access to the participants' world and can help to break down power dynamics between the researcher and researched, encouraging reflection, recall and discussion (Auma et al., 2021).

### 2.3 Sampling

A list of all deprived neighbourhoods in Accra and Ho from the Accra Poverty Mapping Exercise (CHF International, 2010) and United Nations Human Settlements Programme urban profiling report (UN‐HABITAT, 2009) were used to select two neighbourhoods: James Town (Accra) and Dome (Ho) (see further detail in Supporting Information 1). To ensure diversity, participants were purposively selected using quota sampling based on age/reproductive life course stage, gender, body mass index (BMI), education, occupation, maternal status and socioeconomic status (SES) (Supporting Information 2). A subsample (i.e., a third) of the overall study population was randomly invited to partake in the Photovoice study, resulting in 32 participants in Accra and Ho (*n* = 64 total). Recruitment took place through the communities, schools and health facilities (see Supporting Information 3 for additional information).

Before the project began, initial formal meetings with community leaders were held to explain the study and establish community entry. These meetings encouraged community mobilization and engagement with the study and facilitated data collection. RP led the qualitative fieldwork training for seven Ghanaian research assistants. Fieldwork was conducted by native speakers, who were not members of the targeted communities.

### 2.4 Data collection

Data for the Photovoice study were collected between May and December 2017. The Photovoice interview guide was adapted from the original format proposed by Wang (1999) (Supporting Information 4). Initial community engagement activities revealed that women in these urban areas had busy schedules outside of the home setting, making it difficult to organize group discussions at a time suitable to all participants. Therefore, individual interviews were conducted instead of focus group discussions. The Photovoice interview guide was piloted in Accra (*n* = 3) and Ho (*n* = 3) and then amended, accordingly, thus excluding them from the analysis stage.

The Photovoice study took place in three stages. The *first stage* was comprised of an initial home visit, where participants were trained on: (i) the consent process (because they potentially would photograph people); (ii) the Photovoice methodology; (iii) the use of a camera to take photographs; (iv) photography ethics, including the ‘no face or identification details’ protocol to ensure the anonymity of people or places (Supporting Information 5). Participants were asked to take photographs that identify factors driving their dietary behaviours. Specifically, they were asked to take five photographs on the following themes: (i) a place where you eat food and/or drink; (ii) Something that makes eating healthy difficult for you; (iii) something that makes eating healthy easy for you; (iv) something that influences what you eat in your area/neighbourhood; (v) a person that influences your food or drink choices in your area/neighbourhood. During the *second stage*, two follow‐up visits were made to check on progress. The *third stage* consisted of an in‐depth interview that lasted 45–60 min. Interviews were conducted with participants in their preferred language: Ga (*n* = 24); Twi (*n* = 5); English (*n* = 3) in Accra and Ewe (*n* = 28); English (*n* = 3); Twi (*n* = 1) in Ho, respectively. During the interviews, participants told the ‘stories’ related to their five selected photographs. When data collection was complete, a photography exhibition was held to raise awareness of drivers of unhealthy food and beverage consumption in the targeted communities. Photographs from the data collection stages were used as a tool to facilitate dialogue between study participants, the media and local government officers. The photography exhibition also promoted community dialogue and stakeholder engagement by sharing results with the wider community.

### 2.5 Data analysis and synthesis

In‐depth interviews were transcribed and translated verbatim into English for analysis. All coders, RP/AT/SL, used an agreed‐upon codebook in NVivo version 11 to ensure consistency and accuracy, with blind double coding of 25% of the transcripts (Fonteyn et al., 2008). Interviews were coded using deductive (a priori themes) and inductive (data‐driven codes) schemes, to allow for emerging themes (Supporting Information 6). Existing socioecological models of dietary behaviours and systematic review evidence from Africa (Gissing et al., 2017; Story et al., 2008) were used to identify factors, biological, demographic, cognitions (e.g., knowledge and preferences) and practices (e.g., skills and behaviours), influencing dietary behaviours at the individual level. The African Food Environment framework, an expert validated framework created to help prioritize research and intervention development in Africa, was also consulted and used to structure the reporting of our results (Osei‐Kwasi et al., 2021).

Data were synthesized by creating a framework matrix with nodes for different themes and subthemes (Gale et al., 2013). Nodes were then broken down into four populations at different stages of the life course: early adolescents, WRA who were neither pregnant nor lactating, pregnant WRA and lactating WRA. Similarities and differences were highlighted between the different stages and the factors influencing dietary behaviour.

## 3 RESULTS

### 3.1 Sociodemographic characteristics of the study sample

The Photovoice study was conducted with 64 female participants across the two cities (*n* = 32 in Accra; *n* = 32 in Ho). The age range of the study sample was 13–49 years, with 75.0% of participants aged 15–49 years (Table 1). Overall, 37.5% of participants were in work, 12.5% in education and 50.0% were not in work/education. Half of the participants were either pregnant or lactating. Almost half (48.4%) had a BMI ≥ 25 kg/m^2^ (overweight or obese).

**Table 1:** Sociodemographic characteristics of the sample (based on quota sampling)

	Total		Early adolescents 13–14 years		WRA (not pregnant/lactating) 15–49 years		Pregnant WRA 15–49 years		Lactating WRA 15–49 years	
	(*n* = 64)		(*n* = 16)		(*n* = 16)		(*n* = 16)		(*n* = 16)	
	*n*	%	*n*	%	*n*	%	*n*	%	*n*	%
Location										
Accra	32	50.0	8	50.0	8	50.0	8	50.0	8	50.0
Ho	32	50.0	8	50.0	8	50.0	8	50.0	8	50.0
Occupation										
In work	24	37.5	0	0.0	8	12.5	8	12.5	8	12.5
In education	8	12.5	8	12.5	0	0.0	0	0.0	0	0.0
Not in work or education	32	50.0	8	12.5	8	12.5	8	12.5	8	12.5
Household SES^a^										
Lowest SES	32	50.0	8	50.0	8	50.0	8	50.0	8	50.0
Low to middle SES	32	50.0	8	50.0	8	50.0	8	50.0	8	50.0
BMI										
<25 kg/m^2^	33	51.6	8	50.0	9	56.3	8	50.0	8	50.0
≥25 kg/m^2^	31	48.4	8	50.0	7	43.7	8	50.0	8	50.0

^a^ Household socioeconomic status (SES) was measured using the EquityTool (Chakraborty et al., 2016). SES scores were derived using proxy indicators of the household environment (ownership of consumer durables; source of drinking water and type of toilet facilities; type of materials used for the floors and walls; and land ownership). SES quintiles were subsequently derived. Participants were further classified into three groups: lowest SES (first quintile); low to middle SES (second and third quintiles) and high SES (fourth and fifth quintiles). For this project, only participants in the first and second tertiles, representing the lowest and low to middle SES, respectively, were selected.

### 3.2 Individual factors influencing dietary behaviours

**Figure 1:**
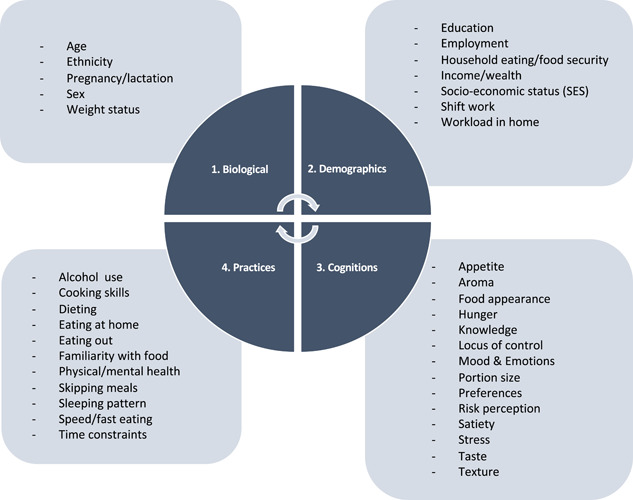
Individual level factors and domains influencing dietary behaviours among early adolescents and women of reproductive age in Accra and Ho, Ghana

Thirty‐eight individual‐level factors were identified to influence dietary behaviour across four domains of biological (5 factors), demographic (8 factors), cognitions (13 factors) and practices (11 factors) (Figure 1). Factors influencing dietary behaviours were similar between early adolescents and WRA, with few marked differences across life course stages.

#### 3.2.1 Biological

Biological factors included age, ethnicity, pregnancy/lactation status, sex and weight status. Eighteen participants referenced their pregnancy/lactating status as an influential factor in their everyday dietary behaviour. Several subfactors used to describe this influence included: medical recommendation, knowledge and willingness to eat what is good for the baby's growth—that is, increased homemade consumption and diversified diet and willingness to consume foods that increase breast milk production: *‘Because if I don't eat a lot or eat healthy food, they [young children] will not get the breast milk to feed on and they need to grow well […] At first, anything I get, I will eat it. But now, I know that I have to feed myself well […] So, I have to eat more vegetable, fruits and also take blood tonic’* (nutrient supplement often rich in iron, Vitamin B12 and folic acid) [Ho, 38 years, lactating, low‐middle SES].

Four participants discussed their weight status and a preference among adolescents and not pregnant/lactating WRA for not being overweight. One 13–14‐year‐old participant explained that her classmate advised her to eat less to lose weight: *‘I was eating a certain food that was not good for me and I was growing fat and she [classmate, 16 years] has been giving me advice of the food that I have been eating […] she helped me so now I am a little bit slim’* [Accra, 14 years, lowest SES]. However, being too thin was considered undesirable and associated with illness. Additional influential factors, such as, ethnicity (*n* = 4) was mentioned by each group, while age (*n* = 1) and sex (*n* = 1) were only discussed by not pregnant/lactating WRA.

#### 3.2.2 Demographics

##### Income, wealth and employment

Demographic factors included income, wealth, employment and household eating/food security. The majority of WRA reported financial barriers (income and wealth) to consistently accessing food, whether it was healthy or otherwise: *‘It is all about having enough money’* [Accra, 35 years, low‐middle SES] (Table 2). Fresh fruit, meat and fish were listed as nutrient‐rich foods that were desirable, but unaffordable: *‘When I have good work to do and the money is coming […] I can buy fresh fish, chicken, I can cook well, buy fruits and eat. But if I don't have work or my work is not going on well, buying food is difficult. So, having a livelihood/income makes it easy to eat healthy’* [Ho, 49 years, lowest SES]. When income was reduced, food provision became difficult. One pregnant participant described coping strategies, such as eating unripe fruit or selecting food items with a reduced price.

**Table 2:** Demographic drivers of dietary behaviours

Factor	Quote	Photovoice image
Theme: Income, wealth and employment		
Employment	‘If I am working all the time, I will get money to be able to buy whatever I want to eat and even eat morning, afternoon and evening. Or it may even be more than the three times a day. So it is good that work all the time’. [Ho, 24 years, lactating, lowest socioeconomic status (SES)]	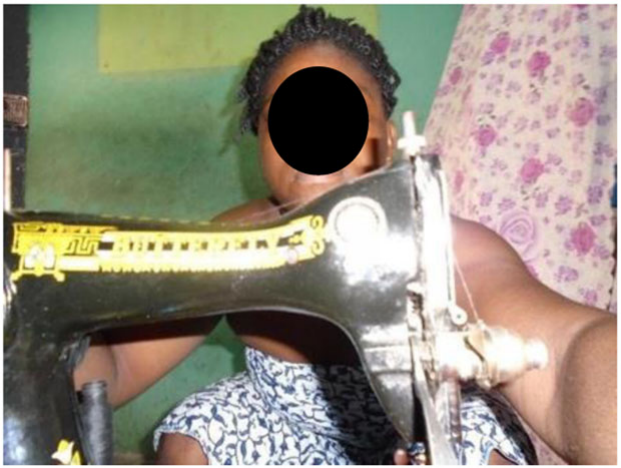
Income/Wealth	‘Because of my pregnancy, I cannot work, so I wait on my husband to send me money before I will be able to eat. At times too, my dad supports me and also my in‐laws support me financially’. [Accra, 19 years, pregnant, lowest SES]	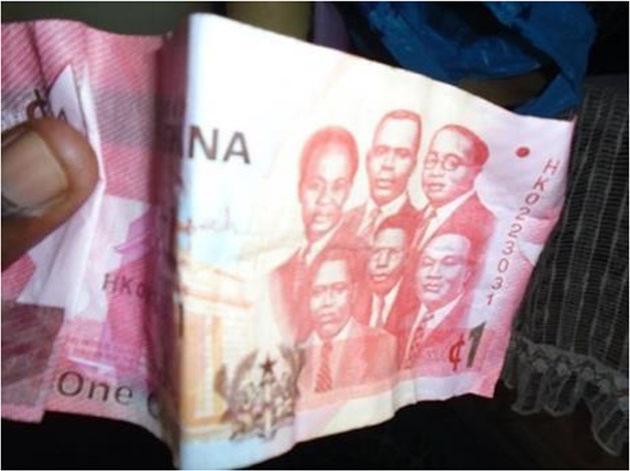
Theme: Household eating/food security		
Household eating	‘These instant noodles that we buy almost every evening, they add all sort of artificial spices to it, the sausage and all those things are not good for our body but we cannot afford the fish. We have no choice than to eat the instant noodles and sausage’. [Accra, 38 years, lowest SES]	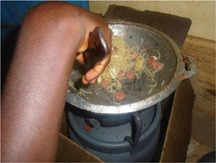
Household food security	‘This is a picture of myself and my friends eating together. We always eat together and that determines what I should eat. I may feel for kenkey and a friend might feel for banku but once I am eating the kenkey, they will join in and eat with me. The same thing happens when they feel for some kind of food and we all eat together. This is because we don't have enough money, so what we have is what we use to buy food and eat together’. [Accra, 19 years, lactating, low‐middle SES]	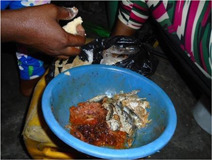

Some adolescents reported going to school and working occasionally. Employed participants in Ho and Accra had similar jobs: ambulatory vendors, market vendors, seamstresses and shift workers (i.e., shopkeepers, waitresses). Participants typically bought more food and cooked at home when their source of income was reliable. Some women reported losing their source of livelihood during pregnancy, which in turn made eating difficult. Participants associated additional sources of revenue with eating enough and eating well. Two pregnant/lactating participants described purchasing sugar‐sweetened beverages when extra money was available. Adolescents mentioned purchasing food, often snacks, with their pocket money.

##### Household eating/food security

Participants aged 15–49 years (not pregnant/lactating) stated a preference for healthy food but reported prioritizing children's dietary needs, as well as school tuition. Among pregnant/lactating participants, eight mentioned serving homemade meals for their families, although this was challenging as they needed to spend money in advance. Two mothers discussed positive dietary behaviours such as sharing fruits (e.g., pineapple, bananas) with their children. However, it was widely observed that income, affordability, time, convenience and seasonality were barriers to eating healthy: ‘fruit is good for us but the prices do change depending on whether they are in season or not and because of that we cannot buy fruits at higher prices’ [Accra, 32 years, lactating, low‐middle SES].

#### 3.2.3 Cognitions

Central cognitions factors included health and nutrition knowledge, risk perceptions around food safety, preferences, food characteristics, hunger, mood and emotions.

##### Health and nutrition knowledge

Study participants seemed to have a relatively good level of health and nutrition knowledge (Table 3). An understanding of how to eat well and stay healthy in these communities was already established in early adolescence. Nutritious diets were identified as rich in fruit, vegetables, green leafy vegetables, eggs, fish and meat. Poor health outcomes were associated with poor quality diets by five participants across the different stages of the life course. As participants were aware that their health was influenced by diet, several referenced dietary changes as a means to control prediagnosed health conditions, such as hypertension. Advice from medical professionals led some women to make positive changes to their diets (e.g., eating more fish than meat because of ‘high cholesterol’): *‘I could fry like 5 to 7 eggs and it was normal for me. But it started having an effect on me. My blood pressure shot up and when I was sent to the hospital, the doctor advised me to stop taking eggs and meat […] So, it's what my heart wants that I eat’* [Accra, 29 years, lactating, lowest SES]. Despite some misconceptions, many participants reported accurate information on nutritious and safe foods.

**Table 3:** Cognitions‐related drivers of dietary behaviours

Factor	Quote	Photovoice image
Knowledge, risk perception and hunger and satiety		
Risk perception	‘When I know how a meal was prepared or the ingredients that were used to prepare a meal, I find it easy to eat the meal but when I do not know how the meal was prepared, I do not find it easy to eat. So, if food is sold close by where we live and I know how it is prepared, I find it easy to eat’. [Accra, 14 years, low‐middle socioeconomic status (SES)]	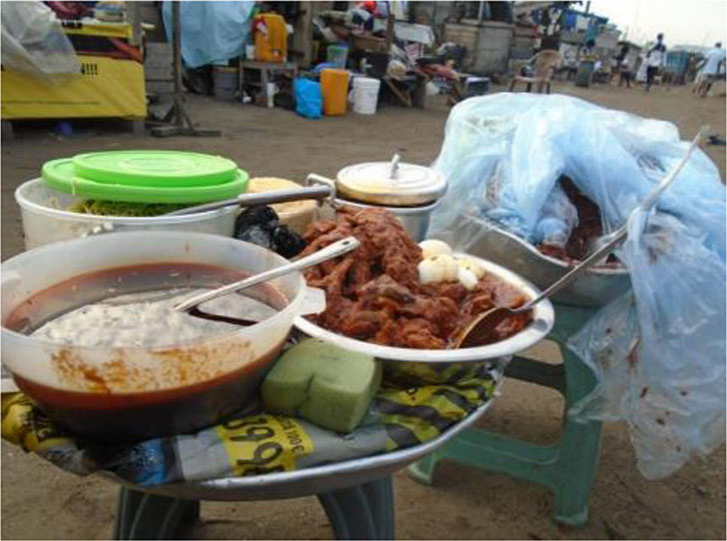
Hunger and satiety	‘The truth is that I usually do not like eating much, I eat very little, so in the mornings, this is the meal I eat. When I eat this meal, I do not feel hungry for a long time during the day. I just drink water’. [Accra, 28 years, low‐ middle SES]	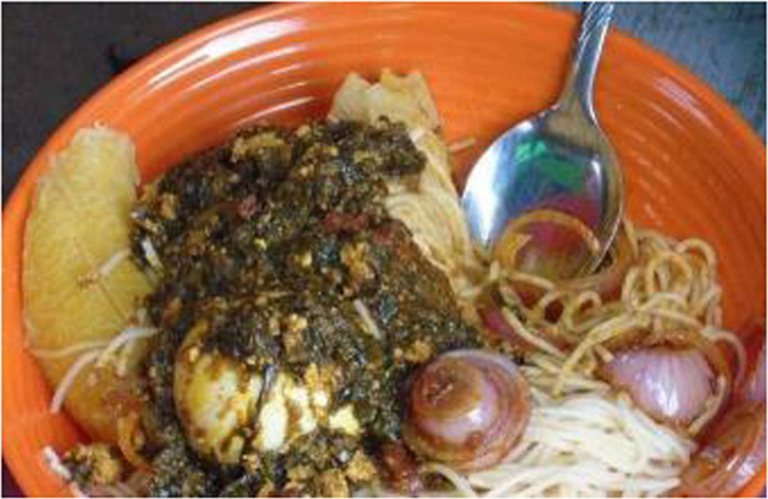

Health and nutrition knowledge and beliefs were quite similar across age groups, with specific pregnancy/lactation‐based examples among those who already had children. Participants who were pregnant described trying to eat food that would: promote foetal/infant growth, encourage good nutritional status and ease the birthing process. General knowledge of healthy eating during pregnancy was good, including, diverse nutrient‐rich local foods and dishes to encourage in‐utero development, such as fresh fruits, yam, fried chicken, palm nut soup, taro leaf (‘nkontomire’) stew and garden egg stew (made with white aubergine). Some pregnant/lactating participants shared correct knowledge of the protective role of fruit, sources of protein and starchy carbohydrates, in addition to some knowledge about the need to avoid sugar, fried instant noodles, salty stock cubes and too much fat. Anaemia during pregnancy was cited frequently as a concern among pregnant women. Eating iron‐rich foods, such as turkey berries (‘kantose’), was frequently recommended to them to prevent anaemia. Reduced consumption of sugar and oil during lactation was advised by family, midwives and other health professionals. Some misconceptions, such as eating ‘too much oil as a cause of malaria’ and the nutrient‐rich composition of malted beverages, frequently consumed during lactation, were held.

##### Risk perceptions around food safety

The majority of participants referenced food safety risks, indicating that there was a lot of anxiety and a good level of knowledge surrounding food safety. In addition, some participants discussed the need to limit the consumption of foods and beverages with additives sold in shops, like sugar‐sweetened beverages and stock cubes. Participants deployed mitigation strategies to avoid falling ill when risk was perceived. Individual hygiene practices such as hand washing before meals were common among pregnant/lactating participants. Washing utensils after eating and preparing food in clean areas were also listed. One 13–14‐year‐old participant mentioned her ability to positively influence food hygiene within her household: ‘Even [I] cook the family food, so due to this I can teach my mom how to be hygienic in cooking’ [Accra, 13 years, low‐middle SES]. Four mothers in the sample specifically cited making food for their children at home to avoid unclean food purchases out of the home.

Two 13–14‐year‐old participants and one lactating participant described avoiding risk by eating food prepared in a hygienic way by someone they know. Some 13–14‐year‐old participants perceived an increased food safety risk when food was prepared outside of the home, purchased as ‘takeaway’ or leftovers from a group event. Some pregnant/lactating participants (*n* = 4) mentioned having a preference for cooking/preparing food at home because they could make food taste the preferred way and ensure hygiene. The location where participants chose to eat was also driven by risk perception. For example, several participants preferred eating indoors. Eating outdoors was viewed as unhygienic, as it was more difficult to avoid house flies and illness. However, several participants preferred eating outdoors to avoid inviting ants or other pests inside the room.

Despite concerns, participants ate outside of the home and often adapted strategies to minimize risk. For example, all participant groups cited looking for clean environments to sit and eat their meals. Buying hot meals and buying from a well‐known, trusted vendor was a key risk mitigation strategy. Food temperature was important; eating food served at a hot temperature to avoid illness was described. Prior experience with food poisoning (stomach ache, diarrhoea, vomiting) made pregnant/lactating participants wary of outside food or unknown vendors. Participants avoided *‘*buying sickness*’* from vendors selling food near gutters and unclean environments. However, despite these strategies, financial barriers sometimes forced participants to buy from unclean vendors. With low incomes and few alternatives, participants frequently mentioned eating whatever they could, if it was cheap and they were hungry: *‘In my area when you don't have money, some of the things you cannot afford, so that will influence you to go in for some maybe cheap food and that will, later on, affect your health’* [Accra, 19 years, low‐middle SES].

##### Preferences

In all groups, food preferences were important. Many participants reported a preference for nutrient‐rich foods such as fish, plantain, fruit and chicken. However, affordability was frequently mentioned as a barrier. Only three participants referenced eating well‐liked foods that were specifically beneficial to their pregnancy. Stable income was also linked with personal and family‐related preferences. This was echoed by some younger and older adolescents, who described eating food available at home and using pocket money to buy sweets and other preferred foods.

##### Food characteristics (texture, taste, aroma, food appearance)

Participants described sensory elements as facilitators or barriers to eating healthily. When the food had a strange taste, look, smell or texture, it was considered unappealing. Younger adolescents specifically mentioned texture, smell, appearance and taste as factors influencing food selection. Some pregnant/lactating participants had a preference for homemade food because they could take personal taste preferences into consideration. One woman indicated that some vendors sold food that was: ‘too sweet’ [Accra, 19 years, pregnant, low‐middle SES]. One 13–14‐year‐old and one 15–49‐year‐old participant described that some vendors used too much ‘kanwe’ in their cooking (i.e., potassium nitrate, for flavour/thickener). Plain rice and small portions of purchased food were considered bland and participants added meat, eggs or fish to create ‘fine’ meals full of flavour. Food appearance, notably in terms of quantity or portion size, was frequently mentioned. Another participant mentioned that freshness was a key element to make a meal taste good. In the pregnant/lactating group, two participants mentioned that sewage inside the home or from toilets or manholes out of the home, made eating difficult and caused loss of appetite.

##### Hunger and satiety

Eating well was associated with feeling full. Eating a filling meal was preferable for the majority of participants, as a way to stay satiated longer: *‘foods like plantain and nkontomire stew and garden egg stew mixed with groundnuts or turkey berries […] these are foods that are healthy for the body. When I eat these foods, I can go the whole day without eating another meal’* [Accra, 16 years, lowest SES]. WRA preferred heavy meals, regardless of pregnancy/lactating status, while other adolescents preferred lighter meals; both mentioning snacking practices and consuming ‘heavier’ meals at home.

Factors in the wider food environment influenced satiety indirectly, such as food prices, affordability and eating a meal alone or without family. In addition, time for work, school and childcare was often brought up as barriers to satiety. Participants described eating more food in a group setting while eating alone was often associated with eating reduced food portions. For example, adolescent participants reported greater satisfaction when eating at home due to the larger portion size. Other participants described needing to drink something (malted drink or sugar‐sweetened beverage) to increase their appetite before a meal, with two WRA taking apple juice and/or eating oranges to stimulate appetite.

##### Mood and emotions

Participants explained that stress and feeling unhappy made eating well difficult. Examples of stress included: school, work, the workload at home and/or unclean food outlets. Being in a calm, relaxed mood facilitated eating. The type of food consumed also influenced individuals’ moods, especially when social connections such as eating with family were considered. Several pregnant/lactating participants and other WRA reported physical cravings for food, including pies, bread and iced kenkey (fermented maize dumpling smoothie with sugar and milk). Some mentioned selecting or changing where they ate to avoid judgment.

#### 3.2.4 Practises

##### Cooking skills

Participants in all reproductive stages described practices such as cooking skills, eating at home or out and time constraints (Table 4). In regard to cooking skills, only one participant (13–14 years) mentioned her ability to cook, with younger participants frequently describing involvement in food preparation or learning cooking skills at home. Cooking at home between one and three times per day was mentioned as a common practice. Many culinary skills were described, such as cooking over charcoal, frying fish, boiling yams and grinding cassava/corn/nuts into flour. Among the 15–49‐year‐old participants, a lack of cooking skills led to unhealthy eating behaviours despite food safety concerns as there were few alternative solutions available: *‘[…] after that experience, you may decide not to buy food there again but because you don't know how to cook at home, you will still go and buy food there again’* [Accra, 19 years, low‐middle SES].

**Table 4:** Practices‐related drivers of dietary behaviours

Factor	Quote	Photovoice image
Cooking skills, eating at home/out and time constraints		
Cooking skills	‘So this one, I do not find it difficult to cook. When I get back from school, I just get it done. And always when I come back from school, they have not cooked so when I enter the kitchen, I just fetch the gari, add the oil and water and then eat, I don't eat again till morning’. [Ho, 13 years, low‐middle socioeconomic status (SES)]	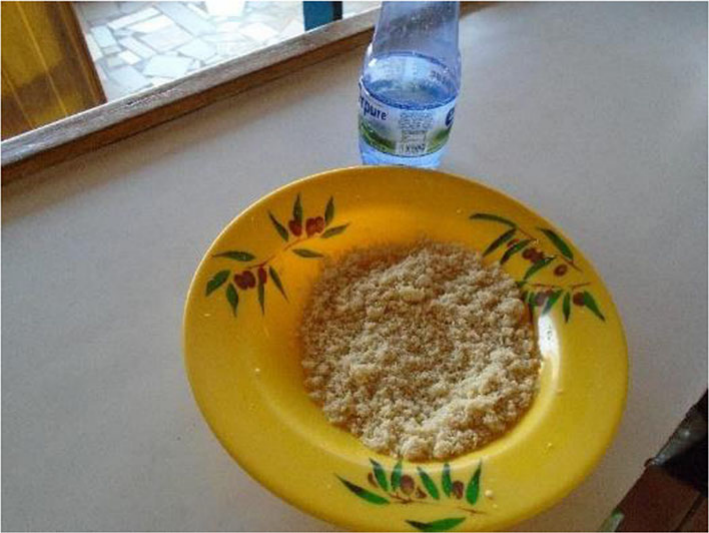
Cooking skills	‘People should buy the fish fresh and prepare it well as home and fry it to their taste and they should eat more fish to be healthy’. [Accra, 16 years, lowest SES]	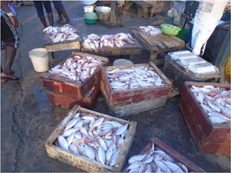
Cooking skills	‘Most of the time, I like preparing my own food at home because I know how and where I will prepare it’. [Accra, 25 years, lactating, lowest SES]	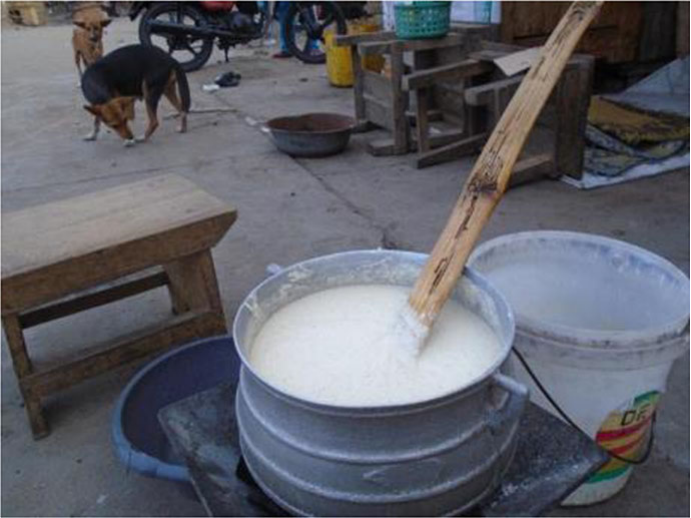
Eating at home	‘Sometimes we eat rice in the afternoon and akple in the evenings or we pound fufu. We all eat the same thing all the time. Whatever I cook, we all eat it together. There is no food that I eat that they don't eat’. [Ho, 27 years, pregnant, low‐middle SES]	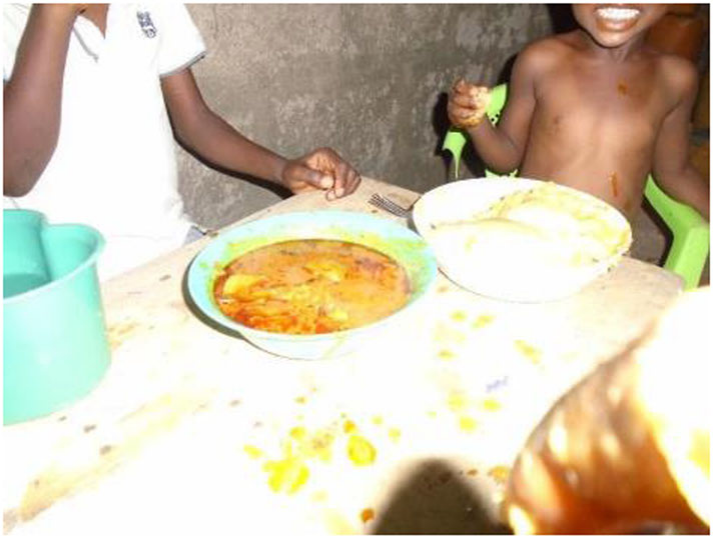
Eating at home	‘So you go and buy outside, you don't know how the person is, you don't know how they take care of their place and how they cook. So when you cook in your own house and eat, it is better than eating outside […] There is no other place that you can eat without getting diseases other than the house’. [Ho, 49 years, lowest SES]	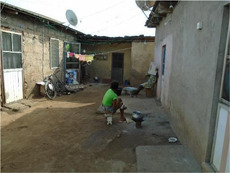
Eating at home	‘[…] Let's say if I have 12 cedis, I can buy onion, tomatoes, pepper, palm oil and fish and I come home to prepare my food. […] I always ensure that I cook most often so that we could have good food to eat in order to avoid problems like stomach ache, diarrhoea and vomiting. This is why I prefer cooking at home’. [Accra, 32 years, lactating, low‐middle SES]	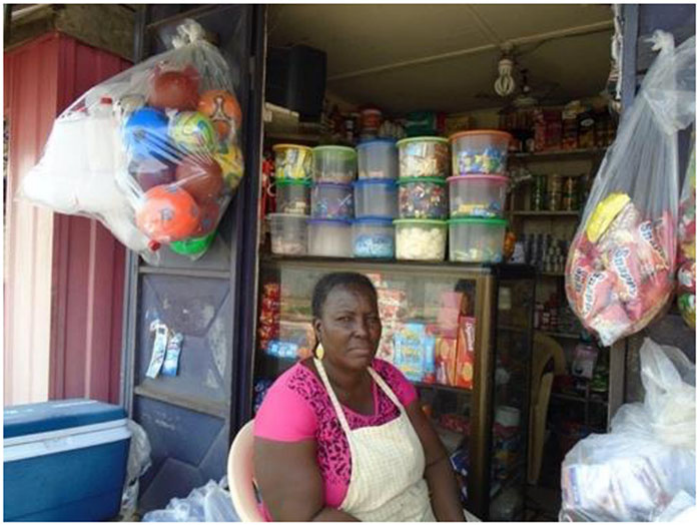
Eating out	‘What makes me buy food from this place is that the woman is clean, she is neat and the food too tastes nice that is why I like buying from her. She sells this food opposite our house’. [Accra, 14 years, low‐middle SES]	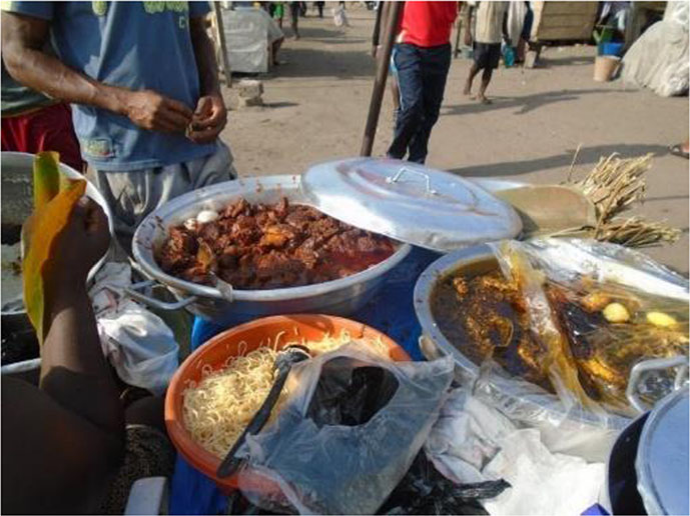
Time constraints	‘I don't eat. Sometimes when I am late to school early in the morning, instead of buying tom brown and bread, I go and buy fried yam and chofi [fried turkey tails] and my friends have been advising me that it is not good, so I should stop’. [Accra, 14 years, lowest SES]	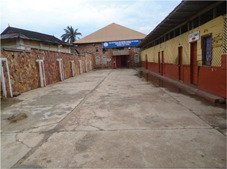
Skipping meals	‘When I go roaming to sell, I find it difficult eating healthy. The picture you see is the head‐pan I carry on my head to sell the Alasa (Africa Star apple). […] When it is like this, I do not get time to eat because I have to come home afterwards, do the household chores and then I start selling soon as I finish the household chores. When I step out, I may or may not find a decent place to buy food. Hence it makes healthy eating difficult for me’. [Accra, 25 years, lactating, lowest SES]	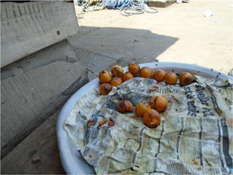
Skipping meals	‘When you have money, you can eat all the time, morning, afternoon, evening. But when there is no money, you can skip it, you can say I won't eat it this morning or I won't eat this afternoon, I'll wait till evening’. [Ho, 24 years, lactating, lowest SES]	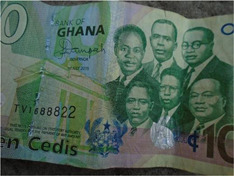
Speed eating	‘[…] So if I get the time, let's say I want to eat fufu in the afternoon and then I happen to get a little time to eat the fufu, I will eat it very very fast, because I also have to go and look at other things. And that is the little time I have to eat the food, so I have to eat it so fast that I would be able to finish early and go and do other things’. [Ho, 22 years, low‐middle SES]	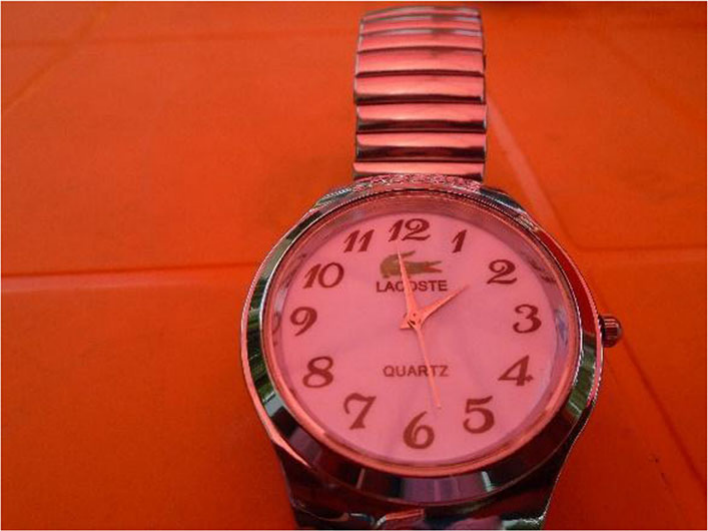

##### Eating at home or out

Eating food prepared at home was most common. Eating out or getting a takeaway were practised with varied frequencies. Homemade food was the overall preference, as ingredients, flavour, portion size and hygiene level could be monitored. Younger adolescents ate out at school canteens, while adults described eating at work or on the way to or from work. Adolescents and WRA reported eating out more often on weekdays and eating more at home on the weekends, influenced by busy school/work schedules and income. Among the 13–14‐year‐old participants, eating homemade food was thought of as a positive, enjoyable practise that facilitated healthy eating. One participant even refused to eat school food. Several participants brought homemade food to work as it was convenient and affordable.

##### Time constraints

*Time constraints* were an overarching theme. Busy lifestyles meant having little free time, which resulted in not 'eating well'; with unbalanced meals/diets at varied frequencies reported. Some adolescents described buying food before school due to time constraints. Among pregnant/lactating WRA, not having enough time to cook influenced the diets of the entire household. For example, participants described buying instant noodles when arriving late/tired from work. Many healthy food and meals, such as cassava soup and plantain‐based meals, were cited as requiring more time and more financial resources to prepare. Some participants stated that they did not have enough time to eat what they wanted. As a result, heavy meals were preferred by many to stay full for longer durations, especially when the reduced time to purchase/make/eat meals (i.e., school, work and childrearing) were considered. Home‐made food was sometimes brought to work/school due to time constraints: *‘I would pour some of the tom browns in the thermos and then take it to work […] and [when] I feel hungry I take some’* [Ho, 38 years, lactating, low‐middle SES].

*Snacking*: Among adolescents, long school days from 7:00 AM–4:00 PM were cited as a barrier to healthy eating. Purchasing snacks during school breaks was frequently cited: *‘During the first break, I take in Kalyppo [fruit drink] and biscuit then during the second break then we eat the canteen food’* [Accra, 14 years, lowest SES]. Snack foods often included sweet foods, such as chocolate, ice cream, candy, biscuits, yoghurt, sugar‐sweetened beverages and fried foods. Three participants linked the frequency of their snacking with their pocket money.

*Skipping meals*: Among older participants, snacking was an alternative to eating a full meal, as time influenced the quantity and timing of meals. Participants tended to skip meals during the day and only ate in the morning or at night. Being too busy with work and not having time to stop and eat was mentioned by several participants, especially shift workers. One lactating woman indicated skipping meals as a barrier to eating healthily.

*Speed eating*: Participants across age groups described eating quickly, despite their stated preference for eating slowly in a comfortable location. Time constraints and workload at home/employment potentially drove participants to eat fast. Caregivers and/or employed women felt rushed when eating, with one participant describing that she had to eat *'*fast fast*'*. Another pregnant participant indicated that when eating with others, she felt a need to eat quickly so that she was able to eat enough: *‘when you are eating with other people, you want to eat quickly and be full before all the food gets finished. Other times too, when you are eating with someone […] they will eat all the meat on the food before you finish eating’* [Accra, 15 years, pregnant, lowest SES].

## 4 DISCUSSION

This study explored individual‐level factors influencing dietary behaviours among adolescent girls and women at different stages of the reproductive lifecycle in urban Ghana. Thirty‐eight factors were identified as having an influence on dietary behaviour across four domains within the individual level: biological (5 factors), demographic (8 factors), cognitions (14 factors) and practices (11 factors). The most frequently cited factors were income/wealth (demographic); nutrition knowledge/preferences/risk perception (cognitive); and cooking skills/eating at home/time constraints (practices). Dietary behaviours were influenced by similar factors, with few marked differences across life course stages. For example, pregnancy/lactating status influenced dietary behaviour through medical advice, awareness and willingness to eat foods that promote infant/child growth and development.

Many individual‐level factors, such as the cost of food, overlapped with the wider social and physical food environments (Pradeilles et al., 2021; Wanjohi et al., 2022). This suggests that interventions need to account for multiple levels and wider drivers of food consumption. This supports findings from previous studies in Kenya (Downs et al., 2022) and Ghana (Boatemaa et al., 2018), showing the need to target multiple levels of the food environment to help women negotiate factors such as food safety, nutrition, time, cost trade‐offs that prevent them from adopting healthy diets.

Despite low levels of education, there appears to be an overall high level of knowledge and awareness of food safety and food hygiene and the impact these may have on diets. Food safety was also observed as a key factor influencing adolescent dietary behaviours in Ethiopia (Trubswasser et al., 2020) and women of reproductive age in Uganda (Yiga et al., 2020). Participants used several risk mitigation strategies, such as preparing and consuming homemade food and eating in clean environments, to ensure the hygienic preparation of food consumed. Despite food safety concerns, participants continued to eat out of home, buying hot meals, eating in cleaner environments and from familiar vendors to reduce perceived food safety risks where finances allowed. Continued eating out practices, influenced by affordability, preference and taste and lack of alternatives were observed in a recent systematic review on food safety concerns in LMICs (Liguori et al., 2022). Actions to improve food safety among food vendors have emerged as a core concern among participants in several research studies in Ghana (Boatemaa et al., 2018; FAO, 2016; Pradeilles et al., 2021; Rheinlander et al., 2008). Individuals' primary concern related to food hygiene is observed in street food practices in LMICs (Akparibo et al., 2021; Alimi, 2016; Omari & Frempong, 2016), with concern increasing among participants that have experienced prior episodes of food‐borne illness (Adam et al., 2014), which supports the need to implement healthy food environment policies in these cities (Laar et al., 2020). Another qualitative study from the *Dietary Transitions in Ghana project*, conducted in the same targeted communities in Accra and Ho, showed that efforts (i.e., research, one‐off events) to address the issue of unhealthy diets among adolescent girls and women exist but are scarce. These were often implemented within school settings, community health centres, churches or mosques (Pradeilles et al., 2019).

Remaining satiated for longer durations was valued as time constraints and the price of eating out of home was challenging. Time constraints emerged as a key factor for eating unbalanced meals and overall diets. Participants reported being too busy to eat as much or as often as they liked, often skipping meals or eating ‘on the go’. In addition, adolescents attending school preferred quick meals, such as snacks. This could lead to increased consumption of energy‐dense, nutrient‐poor foods, which are widely consumed among this group, without creating a feeling of satiety (Drewnowski & Darmon, 2005). Among low SES groups, there appears to be an emphasis on consuming maximum calories, rather than nutritional quality (Darmon & Drewnowski, 2008). A study in the same communities found that time allocated to a meal was usually <30 min for the vast majority of study participants (Holdsworth et al., 2020). This finding supports increased incentives and subsidies targeting local food vendors to provide healthy foods that are convenient and can be consumed quickly (Holdsworth et al., 2020).

This study includes several strengths, namely, the sampling method used to achieve diversity across the life course, application of the African Food Environment framework and the use of Photovoice (i.e., added value over commonly used methods like in‐depth interviews and focus group discussions only). Participants were asked to tell their stories and to engage with a research topic that sought to better understand the current situation within their community. Using photography allowed participants in low‐income communities to have an additional means of communication to identify, capture and discuss challenges and facilitators to eating healthily. While individual Photovoice interviews were conducted in place of group discussions, participants were able to discuss their concerns with a larger audience directly during a community‐based photography exhibition (Pradeilles et al., 2021). Nevertheless, as participants were only selected from two neighbourhoods in Accra and Ho, additional or differing factors may also be a concern within urban neighbourhoods and rural areas in Ghana. It is also important to consider the potential for limited success when individual‐level approaches do not account for the wider food environment factors that influence the individual level (Allender et al., 2015; Doak et al., 2006; Mackenbach et al., 2014; Osei‐Kwasi et al., 2020; Story et al., 2008; Tanentsapf et al., 2011).

## 5 CONCLUSION

In conclusion, investigating the individual‐level factors that influence dietary behaviours through a Photovoice study demonstrated that there is a wide diversity of individual‐level factors, such as affordability, food safety concerns and time constraints, that should be considered when designing interventions to promote healthy eating practices. These factors are linked with the wider food environment, which reflects the complexity of factors influencing dietary behaviours. Our findings suggest that the life course stage, particularly for adolescents and WRA, had less influence on overall diet quality than socioeconomic barriers or food safety concerns. Hence, interventions should focus on providing the means to achieve a healthy diet to all adolescents and WRA in deprived urban areas in Ghana. Redesigning fiscal and physical food environment policies would help support access to a healthy diet for all. Further implementation research is needed to ensure that individual‐level factors are considered in interventions and policies that improve food environments, especially as promoting energy‐dense, nutrient‐rich diets in deprived urban settings remains an unresolved challenge.

## AUTHOR CONTRIBUTIONS

Michelle Holdsworth, Amos Laar, Francis Zotor, Hibbah A. Osei‐Kwasi, Nicolas Bricas, Paula Griffiths, Robert Akparibo, Richmond Aryeetey, Rebecca Pradeilles designed the research study and contributed to protocol development. Akua Tandoh/Senam Klomegah contributed to protocol development and led the data collection and translation/transcription of interviews. Akua Tandoh, Senam Klomegah and Rebecca Pradeilles coded the data. Julia Liguori analysed and synthesised the data with support from Agnès Le Port, Michelle Holdsworth and Rebecca Pradeilles. Julia Liguori, Rebecca Pradeilles, Michelle Holdsworth and Agnès Le Port wrote the first draft of the paper. All authors reviewed the manuscript and approved the final version.

## CONFLICT OF INTEREST

The authors declare no conflict of interest.

## ETHICS STATEMENT

Ethical approval for the study was received from the Ghana Health Service Ethics Review Committee (GHS‐ERC 07/09/2016) and the University of Liverpool (1434 25/1/2017). The University of Sheffield Research Ethics Committee and Loughborough University's ethics Committee recognized the ethical review processes of the Ghana Health Service Ethical Review Committee and, therefore, did not require additional independent ethical review. Written informed consent was obtained from participants aged ≥18 years and consent of legal guardians of participants aged 13–17 years. All minors (participants aged 13–17 years) provided assent before being interviewed. Participants used a photograph release form to request consent/assent if a person's face was visible in a photograph. Participants granted permission for photograph re‐use in scientific outputs. The ethical committee also granted permission for photograph re‐use in scientific outputs.

## Supporting information

Supporting information.

## Data Availability

An open access data repository, DataSuds (part of the Dataverse), was used to write this article. Metadata and tools for Accra and Ho can be accessed here: https://doi.org/10.23708/XSACNA.
