# Supplementary material for: Individual‐level drivers of dietary behaviour in adolescents and women through the reproductive life course in urban Ghana: A Photovoice study
Source: Matern Child Nutr. 2022 Aug 8;18(4):e13412. doi: 10.1111/mcn.13412 (PMC9480960; doi:10.1111/mcn.13412)
Supplement: Supplementary file 1 — Supporting information. [file MCN-18-e13412-s001.docx]

**Supplementary Appendix 1: Selection of neighbourhoods in the two cities in Ghana**

In Accra, the selection of a neighbourhood was informed by the *Accra Poverty Mapping Exercise* (CHF International, 2010). Four areas were identified as being poverty endemic. Amongst these, Ga Mashie which is comprised of James Town and Ussher Town, was purposively selected as it was an area the research team could feasibly work in. A simple random sampling exercise was then applied, and James Town was selected as the neighbourhood of interest. In the city of Ho, the United Nations Human Settlements Programme (UN-HABITAT 2009) urban profiling report informed the selection of the study site. The report highlighted that 36% of the population lived in four poor areas within the city: Bankoe, Hliha, Ahoe and Dome (United Nations Human Settlements Programme, 2009). Amongst these four areas, Dome was then randomly selected.

**References**

CHF International. (2010). Accra Poverty Map: A guide to urban poverty reduction in Accra. Retrieved from: <https://www.globalcommunities.org/node/37186>

United Nations Human Settlements Programme. (2009). Ghana: Ho city profile. Retrieved from: <https://uni.unhabitat.org/wp-content/uploads/2014/07/Ghana-Ho-City-Profile.pdf>

**Supplementary Appendix 2: Quota sampling plan**

**Table 2a: Quota sampling plan for the Photovoice activity in Accra**

|  | | | | | | | | | |
| --- | --- | --- | --- | --- | --- | --- | --- | --- | --- |
| **SES**  **Reproductive**  **Life Course** | Lowest SES | | | | Low to middle SES | | | | **N** |
| 13-14y (not pregnant or  lactating) | Not in work or education | | In work or education | | Not in work or education | | In work or education | | 8 |
|  | BMI<25 | BMI≥25 | BMI<25 | BMI≥25 | BMI<25 | BMI≥25 | BMI<25 | BMI≥25 |  |
| 15-49y (not pregnant or lactating) | Not in work or education | | In work or education | | Not in work or education | | In work or education | | 8 |
|  | BMI<25 | BMI≥25 | BMI<25 | BMI≥25 | BMI<25 | BMI≥25 | BMI<25 | BMI≥25 |  |
| 15-49y (pregnant) | Not in work or education | | In work or education | | Not in work or education | | In work or education | | 8 |
|  | BMI<25 | BMI≥25 | BMI<25 | BMI≥25 | BMI<25 | BMI≥25 | BMI<25 | BMI≥25 |  |
| 15-49y (lactating) | Not in work or education | | In work or education | | Not in work or education | | In work or education | | 8 |
|  | BMI<25 | BMI≥25 | BMI<25 | BMI≥25 | BMI<25 | BMI≥25 | BMI<25 | BMI≥25 |  |
| **Total sample** | | | | | | | | | **n=32** |

**Table 2b: Quota sampling plan for the Photovoice activity in Ho**

|  | | | | | | | | | |
| --- | --- | --- | --- | --- | --- | --- | --- | --- | --- |
| **SES**  **Reproductive**  **Life Course** | Lowest SES | | | | Low to middle SES | | | | **N** |
| 13-14y (not pregnant or  lactating) | Not in work or education | | In work or education | | Not in work or education | | In work or education | | 8 |
|  | BMI<25 | BMI≥25 | BMI<25 | BMI≥25 | BMI<25 | BMI≥25 | BMI<25 | BMI≥25 |  |
| 15-49y (not pregnant or lactating) | Not in work or education | | In work or education | | Not in work or education | | In work or education | | 8 |
|  | BMI<25 | BMI≥25 | BMI<25 | BMI≥25 | BMI<25 | BMI≥25 | BMI<25 | BMI≥25 |  |
| 15-49y (pregnant) | Not in work or education | | In work or education | | Not in work or education | | In work or education | | 8 |
|  | BMI<25 | BMI≥25 | BMI<25 | BMI≥25 | BMI<25 | BMI≥25 | BMI<25 | BMI≥25 |  |
| 15-49y (lactating) | Not in work or education | | In work or education | | Not in work or education | | In work or education | | 8 |
|  | BMI<25 | BMI≥25 | BMI<25 | BMI≥25 | BMI<25 | BMI≥25 | BMI<25 | BMI≥25 |  |
| **Total sample** | | | | | | | | | **n=32** |

**Supplementary Appendix 3: Recruitment strategy**

To identify eligible participants, a screening questionnaire was administered to participants using electronic data capture (Samsung Galaxy tab-4), in order to obtain information relating to socio-demographic characteristics (i.e. place of residence; date of birth; weight and height for the calculation of body mass index; education; occupation and SES). SES was measured using the EquityTool which is a short, validated and country-specific tool to measure wealth (Chakraborty, Fry, Behl, & Longfield, 2016). Household SES scores were derived from 13 questions: ownership/facilities (colour television, refrigerator, video deck/DVD/VCD, bank account, electricity, wall clock, cabinet/cupboard, type of fuel used for cooking, household toilet facility, drinking water source, agricultural land, household floor and exterior walls material). Household scores were then compared to the average scores for urban Ghana and SES quintiles were subsequently derived. Participants were further classified into three groups: lowest SES (1st quintile); low to middle SES (2nd and 3rd quintiles) and high SES (4th and 5th quintiles). For this project, only participants in the 1^st^ and 2^nd^ tertiles, representing the lowest and low to middle SES respectively were selected.

The screening tool was then imported into CSPro version 6.3 and piloted using electronic data-capture. The screening tool was revised accordingly after piloting.

**References**

Chakraborty, N. M., Fry, K., Behl, R., & Longfield, K. (2016). Simplified asset indices to measure wealth and equity in health programs: A reliability and validity analysis using survey data from 16 countries. *Global Health Science and Practice*, *4*(1), 141–154. https://doi.org/10.9745/GHSP-D-15-00384

**Supplementary Appendix 4: Photovoice Interview Guide**

**Presenting the photovoice exercise to participants**

Good morning/afternoon (name of participant), we are here to discuss the photovoice activity we told you about during the 24hr recall data collection a few weeks ago.

What is Photovoice?

A process of collecting information and expressing issues and concerns through photos. Photovoice asks participants to use photographs to record aspects of their lives and experiences.

In this project ***(DIETARY TRANSITIONS IN GHANAIAN CITIES)***, we will be asking you to take some pictures of the things that make you choose the foods and drinks you consume at home or elsewhere (e.g., people you relate with, your environment, and any other things). We are doing this, so that we can help the formulation of policies that improve diets.

We will give you a digital camera which you will use to take photographs on some topics we have identified in a topic list below.

The pictures you will take and the discussion we shall have with you on your pictures, will help us understand how our family members, friends, neighbours, the places we stay in and the places we go, affect or influence the foods we eat and drink.

Furthermore, these pictures will help us to find ways that could make women and adolescent girls in Ghana eat and drink more healthily. To this end we would like to share some of the photographs that you and the other women in this project take in an exhibition later in the project so that people who make decisions about food and drink in this neighbourhood can have a better idea of the things that influence the foods you eat and drink. Your pictures can therefore be powerful in making change!

This Photovoice exercise will be in two sessions.

Today, we will take you through the picture list and how to use the camera. We will show you how to turn the camera on/off, how to snap the pictures and how to check if the pictures are on the camera or not. Then we will go through the picture list.

There are some few things to note:

**Photo Ethics and Safety**

- If you take a picture and the face of person shows, you will need to ask permission from the person before the picture can be used. We will give you a ‘Photo Release’ Form to use to get permission.
- Though we want you to take these pictures for us, please ensure you are safe anytime you go out to take pictures.
- You can go with another person to take the pictures.
- Don’t do anything you wouldn’t usually do.
- Don’t go anywhere you wouldn’t usually go.
- Please do not enter into a person’s private space to take your pictures.
- Avoid disclosure of embarrassing facts about individuals through your pictures.
- Avoid taking pictures of people in such a way that leaves a negative and inaccurate impression about that person.

In the second part, we will come after 7 -10 days for the camera, have the pictures printed and then come back for a discussion on the pictures. During the 7-10 days, we will check in to see how you are doing and check the progress of the picture taking, as well as answer any questions you have with regards to the picture list or the camera, we gave you.

Let's start with showing you how to use the camera and after, we will go through the picture list. Please let us know if you do not understand or something is not clear. We will take you through it till everything is clear.

**PICTURE LIST**

Please try and take at least one picture that shows each of the five headings/topics below

1)  A place where you eat food and/or drink

2) Something that makes eating healthy difficult for you (for example, time; price of food/drink; home; work; school; neighbourhood/community; restaurants and fast food outlets; supermarkets; convenience and corner store).

3) Something that makes eating healthy easy for you (for example, time; price of food/drink; home; work; school; neighbourhood/community; restaurants and fast food outlets; supermarkets; convenience and corner store).

4) something that influences what you eat in your neighbourhood/community (for example, income/money; home; work; school; neighbourhood/community; restaurants and fast food outlets; supermarkets; convenience and corner store).

5) A person that influences your food or drink choice in your area (i.e. family; friends; peers; other)

​​

**INTERVIEW PROTOCOL**

1. Ask Participants to return with SD card after 7-10 days
2. Print photographs for interview
3. Match photo numbers with photo topics for data storage and identification on the day of interview.

Remind participant

- 1. In this project, we are trying to understand the things that make you choose the foods and drinks you consume at home or elsewhere (e.g. people you relate with, your environment, and any other things).
  2. We are doing this, so that we can help the formulation of policies that improve diets.

1. Start qualitative interview (see guide below) using pictures as focus for the interview

**Photovoice: Qualitative Interview Questions**

- Can you pick the most important picture to you please?
  1. Why have you chosen this picture over other pictures you took to include?
  2. Can you tell me about what this picture shows? *(note: try and get a thorough description of the photos and make sure the participant has covered why the picture is important to understanding food choices in their daily life).*
  3. If you had to tell one sentence to tell the story of this picture to a person looking at it in an exhibition what would it be?
- Which picture do you want to talk about next? *(note: use this prompt for all other pictures left to discuss)*
- For each picture:
  1. Can you tell me about what this picture shows? *(try and get a thorough description of the photos).*
  2. Can you tell me why this picture is important to understanding your food choices in your daily life? *(ask this only if this has not been covered in the description of the picture above).*
- If you could choose a photo to appear in an exhibit to tell a story about the food and drink environment in your community (good or bad things), which one would it be and why? *(note: If photo contains a face, check a photo release form exists, if not, choose another picture)*
- Can you describe any photos you would like to have taken to tell us about food choices in your neighbourhood/community that you were not able to take? What stopped you being able to take these?
- Ask participant to say (or write) a short caption/sentence that best describes what their picture is showing *(note: only ask this if the participant did not come up with a caption/one sentence description when discussing this particular photo earlier).*

1. Debrief - What is your general feeling about this activity?
   1. Did you learn anything from this activity e.g. about what influences consumption of food and drink in your community?
   2. What was your experience with learning to handle the camera?
   3. Did you feel safe handling the camera outside?
   4. Do you have any suggestions for improving the process?

**Supplementary Appendix 5: Ethics and safety guidelines for Photovoice**

**Ethics and Safety**

- If you take a picture and the face of person shows, you will need to ask permission from the person before the picture can be used. We will give you a ‘Photo Release’ Form to use to get permission.
- Though we want you to take these pictures for us, please ensure you are safe anytime you go out to take pictures.
- You can go with another person to take the pictures.
- Don’t do anything you wouldn’t usually do.
- Don’t go anywhere you wouldn’t usually go.
- Please do not enter into a person’s private space to take your pictures.
- Avoid disclosure of embarrassing facts about individuals through your pictures.
- Avoid taking pictures of people in such a way that leaves a negative and inaccurate impression about that person.

**Supplementary Appendix 6: template codebook for Ghana for the individual-level environment**

| **Name of nodes** | **Description** |
| --- | --- |
| **Biological** |  |
| Age |  |
| Health & sickness status |  |
| Pregnant, lactating |  |
| Sex |  |
| **Cognitions** |  |
| Attitudes |  |
| Body image perceptions | e.g., Eating French fries makes you grow fat |
| Familiarity | e.g., Not being familiar with water in Accra causes stomach problems, whilst drinking water in Ho does not cause stomach problems. |
| Feelings (both physical and emotional) | e.g., I feel like vomiting when I eat kenkey |
| Health and nutrition knowledge & beliefs | Health awareness; health benefits, perceptions of certain foods (e.g., fruits and vegetables are detoxifiers, kenkey and oats can give you strength, increase blood levels by eating nkontomire stew regularly, eating French fries makes you grow fat); knowledge of what a healthy or balanced diet is; knowledge of benefits of certain nutrients (e.g., meat is a good source of protein); foods that are good or bad for your body. |
| Hunger & satiety |  |
| Lack of appetite | e.g., I always had a poor appetite, personally getting me to eat plenty of food is difficult. I don't know why but as a person I don't like eating a lot of food. |
| Loneliness |  |
| Mood & Stress |  |
| Preferences: (un)favourable; (un)likeable foods and meals | e.g., I eat chips for lunch because it is light and I don't eat heavy food like ugali for lunch |
| Preferences: cooking & preparation |  |
| Preferences: foods that can be eaten with the rest of the family |  |
| Preferences: Place(s) to eat | e.g., Favourite restaurant |
| Preferences: sourcing & provisioning |  |
| Preferences: variety of ways to cook and eat the food |  |
| Taste & Aroma | e.g., The stew is too sweet and the rice too is not cooked well; when I buy food from this place, I find it difficult eating. If there is no food vendor open at the time I want to buy food and I go and buy from this place, it is hard for me to eat. |
| Values |  |
| Practices |  |
| Cooking and preparation |  |
| Eating alone or together |  |
| Eating cold or hot food |  |
| Eating from a shared bowl |  |
| Eating homemade foods & meals |  |
| Eating home-packed foods & meals | e.g., Bringing food from home to eat at work |
| Eating in small or large quantities |  |
| Eating light or heavy foods or meals |  |
| Eating or drinking out, outdoors | e.g., Eating outside might predispose to diseases due to unsanitary conditions; eating out is not healthy as you do not get the right nutrients and I also get runny stomach. |
| Eating quickly or slowly |  |
| Eating standing or sitting |  |
| Eating or drinking in, indoors | e.g., Eating indoors allows to not rush the eating process; e.g. I feel comfortable eating in my room. Because when I am eating here, there will be some people outside looking into your face. They just want to know how you eat, so I like eating in the room always. |
| Skipping meals |  |
| Snacking |  |
| Sourcing and provisioning |  |
| Time & convenience | e.g., Prefers pojo over vegetables because cooking time is shorter. e.g., lengthy preparation time (long time to clean offals). e.g., lengthy cooking time.  e.g., sometimes when I want to eat, maybe I want to learn as well, so I have to reduce the time I will use to eat and go and learn. |
| Skills |  |
| Cooking skills |  |
| Socio-demographic |  |
| Education |  |
| Employment |  |
| Ethnicity & tribe |  |
| Household food expenditure |  |
| Marital status |  |
| Wealth | e.g., Food stuff that you can buy and use to cook that will give you strength. Also you can use it to get good things to buy. e.g., when you have money you can buy whatever you want to buy. You won't have to go and ask money from anybody. You go and buy whatever you want and come home and cook.  e.g., whatever you have to buy, so far as you have money, you can buy it. e.g., without money you cannot buy food and eating healthy is difficult. |

Note: This codebook only includes the nodes and sub-nodes for the individual-level of the socio-ecological framework we used to guide the analysis. However, the full codebook covers all four levels of the socio-ecological framework: i. individual (e.g., preferences, knowledge, socio-demographic characteristics); social (e.g. family, friends and peers); physical (refers to environments in which people eat or source food, including the home, workplace, schools, restaurants and supermarkets) and macro (e.g. food marketing, food production and distribution systems).
